# Parotid salivary duct carcinoma: a single institution’s 20-year experience

**DOI:** 10.1007/s00405-019-05454-0

**Published:** 2019-05-06

**Authors:** Dominik Stodulski, Bogusław Mikaszewski, Hanna Majewska, Jerzy Kuczkowski

**Affiliations:** 1 Department of Otolaryngology, Medical University of Gdańsk, ul. Smoluchowskiego 17, 80-214 Gdańsk, Poland; 2 Department of Pathomorphology, Medical University of Gdańsk, Gdańsk, Poland

**Keywords:** Salivary duct carcinoma, Parotid, Survival rates, Prognostic factors

## Abstract

**Purpose:**

The aim of the study was to assess the treatment results of the parotid gland salivary duct carcinoma (SDC).

**Material and methods:**

A retrospective clinicopathological analysis of 40 patients treated for parotid SDC in 1996–2015 was performed. The impact of following factors on 5-year disease-free survival (DFS) and overall survival (OS) was studied: age, sex, preoperative 7th nerve palsy, skin infiltration, pT, pN, surgical margin, type of parotidectomy and neck dissection, histology (SDC de novo vs. SDC ex pleomorphic adenoma, SDCexPA), intra/periparotid lymph nodes metastases, perineural invasion (PNI), extraparenchymal extension (EPE), and overexpression HER2.

**Results:**

The average age of the patients was 62 years (ranged from 39 to 81). Males predominated (57.5%). Patients with the clinical stage IV predominated (82.5%). In 1/3 of patients preoperative, 7th nerve palsy occurred. All patients were treated surgically, and all but one had supplementary radiotherapy. In 28 patients (70%), total radical parotidectomy was performed. A neck dissection was performed in all patients. In 19 cases (47.5%), SDCexPA was diagnosed. Negative microscopic surgical margin was obtained in 60% of patients. The follow-up for the whole analyzed group ranged from 2 to 22 years, average was 11.6 years. In 23 patients (57.5%), the disease recurred. Local recurrence was observed in 10 (25%) and distant metastases in 15 (37.5%) cases. 20 patients (50%) died of cancer. 5-year DSF and OS were 42.5% and 41%, respectively. Univariate analysis proved that the significant influence on the survival had 7th nerve palsy (*p* = 0.024 and *p* = 0.017, respectively), higher pT-stage (*p* < 0.001), radical parotidectomy (*p* = 0.024 and *p* = 0.022), radical treatment of the neck (*p* = 0.001 and *p* = 0.002), EPE (*p* = 0.040 and *p* = 0.028), and histology SDCexPA and PNI (*p* = 0.036 and 0.048). Multivariate analysis showed that independent prognostic factors were the 7th nerve palsy and the histology SDCexPA, which worsened 5-year DFS, respectively, 3.61 and 3.94 times (*p* = 0.033 and *p* = 0.026). On the other hand, on 5-year OS, only 7th nerve palsy had an influence (3.86 times worse prognosis, *p* = 0.033).

**Conclusions:**

SDC is a clinically aggressive cancer with high risk of local recurrence and distant metastases, however, with a chance of curing of around 40%. In the majority of patients, a radical surgical treatment is necessary due to the high clinical stage of disease. Worse prognosis have patients with preoperative 7th nerve palsy and in whom SDC develops in pleomorphic adenoma.

## Introduction

Salivary duct carcinoma (SDC) was described by Kleinsasser in 1968 [1]. It is an aggressive tumor similar to high-grade mammary ductal carcinoma [2]. SDC may develop de novo or within the pleomorphic adenoma (salivary duct carcinoma ex pleomorphic adenoma, SDCexPA) [3]. SDC represents < 1.8% of all major salivary gland tumors and about 10% of all salivary gland malignancies [4]. It is, therefore, a relatively rare cancer; however, its occurrence may be underestimated in patients with tumors classified as carcinoma ex pleomorphic adenoma [3, 5]. Clinical series from single centers showing SDC count from several to several dozen patients are an important source of information about this cancer, as each case report provides new or confirms known information about its clinical course and prognosis. In most cases, prognostic factors are presented in general for all salivary glands [6–12]. Due to differences in symptomatology and treatment between individual major salivary glands, the authors decided to present their own SDC located only in the parotid gland.

## Patients and methods

The study was in accordance with the Declaration of Helsinki on biomedical research involving human subjects. In 1996–2015, 159 patients with primary parotid gland carcinoma were treated in the Department of Otolaryngology, of which 40 (25.1%) were diagnosed with salivary duct carcinoma (de novo or in pleomorphic adenoma). In all the cases, histological diagnosis was confirmed according to the WHO classification of salivary gland tumors from 2017 [3]. To assess the risk factors for treatment failure, the following variables were examined: age, sex, the presence of preoperative facial nerve palsy, skin infiltration and other symptoms of malignancy, pT-stage (T1 + T2 vs. T3 + T4a), pN-stage (N0 vs. N + ), type of parotidectomy (conservative/semiconservative vs. radical), type of neck dissection (selective vs. radical/modified radical), surgical margin (resection *R*0 vs. *R*1/*R*2), histology (SDC vs. SDCexPA), extraparenchymal extension (EPE), perineural invasion (PNI), status of intra/periparotid lymph nodes (pN- vs. pN + ) and overexpression of *human epidermal growth factor receptor 2* (HER2).

### Treatment protocol

The overriding priority of surgical treatment was the radicality of the procedure, however, whenever there was a possibility of maintaining the continuity of the VII nerve, a conservative parotidectomy was performed. In the cases of enlarged lymph nodes (cN + ) or cT4aN0, MRND/RND was always performed. Selective ND was performed in patients without clinically enlarged lymph nodes and with T1–T3 stage.

### Statistical analysis

All calculations have been carried out by means of Microsoft Excel spreadsheet and STATISTICA, StatSoft, Inc. Ver. 12.0. Statistical package (data analysis software system). The cumulative proportions of patients’ disease-free survival (DFS) and overall survival (OS) were estimated with Kaplan–Meier curves. Log-rank test was used to test the significance of differences in survival between compared groups. Hazard ratios for patients in each subgroup were estimated using a Cox proportional hazards model. For multivariate survival rate analysis, a regression of proportional Cox hazard was used. The statistical significance level was established at *P* < 0.05.

## Results

In the studied group of patients, there were 17 women (42.5%) and 23 men (57.5%), aged from 39 to 81 years (average 62). Patients gave the presence of a parotid tumor lasting from 1.5 to 480 months (median 30 months). In 30 out of 40 patients (75%), there were symptoms indicating the malignant nature of the tumor such as: pain (42.5%), facial nerve palsy (32.5%), rapid tumor growth (35%), cervical lymphadenopathy (20%) and skin infiltration/ulceration (15%). The duration of malignancy symptoms ranged from 1 to 12 months (median 5 months). The local progression (pT) based on TNM from 2017 was as follows: T1-1 (2.5%), T2-9 (22.5%), T3-7 (17.5%) and T4a-23 (57.5%). The pN-stage, on the other hand, was: N0-13 (32.5%), N1-4 (10%), N2b21 (52.5%) and N3-2 (5%) [6]. As many as 33 out of 40 patients (82.5%) were in the IV clinical stage, 6 (15%) in III and only 1 person in II (2.5%). All patients were treated surgically, and with the exception of one they received complementary radiotherapy. 28 patients (70%) underwent total parotidectomy with resection of the facial nerve, 5 (12.5%) total or lateral parotidectomy with preservation of some branches of the facial nerve (semiconservative), and in the remaining 7 cases (17.5%)—the lateral conservative. A neck dissection was performed in all patients. In half of the cases, a radical or modified radical neck dissection was performed, while in the remaining 20 (50%)—selective with II, III, V or in two cases II and III lymph node group. Histologically, in 19 cases (47.5%), SDCexPA was diagnosed. In 13 patients (32.5%) overexpression of the HER2 occurred. A negative surgical margin was obtained in 24 cases (60%), however, in 6 of them it was close (from 1 to 3 mm). In 15 patients (37.5%), the excision was microscopically positive, and in 1—macroscopically (infiltration of the parapharyngeal space). The follow-up for the whole analyzed group ranged from 2 to 22 years, average 11.6 years. Clinical and pathological data are presented in Table 1.

**Table 1:** Clinicopathological features of 40 patients with parotid *salivary duct carcinoma*

Clinicopathological features	*N* (%)
Age (years)	39–81 (mean 62)
Sex	
Men	23 (57.5%)
Women	17 (42.5%)
Signs and symptoms of malignancy	30 (75%)
Facial nerve palsy (preoperative)	13 (32.5%)
Skin infiltration/ulceration	6 (15%)
Pain	17 (42.5%)
Tumor rapid grown	14 (35%)
Cervical lymphadenopathy	8 (20%)
Time of tumor presence (months)	1.5–480 (median 30)
Time of lasting symptoms of malignancy (months)	1–12 (median 5)
Parotidectomy	
Radical (total)	28 (70%)
Semiconservative (total and lateral)	5 (12.5%)
Conservative (lateral)	7 (17.5%)
Neck dissection	40 (100%)
Selective	20 (50%)
Modified radical	11 (27.5%)
Radical	9 (22.5%)
Resection	
R0/R0 (close margin)	24 (60%)
R1	15 (37.5)
R2	1 (2.5%)
Complementary radiotherapy	39 (97.55)
pT-stage	
T1	1 (2.5%)
T2	9 (22.5%)
T3	7 (17.5%)
T4a	23 (57.5%)
pN-stage	
N0	13 (32.5%)
N1	4 (10%)
N2(b)	21 (52.5%)
N3	2 (5%)
Clinical stage	
I	0
II	1 (2.5%)
III	6 (15%)
IVA	32 (82.5%)
Histology	
SDC	21 (52.5%)
SDC EX PA	19 (47.5%)

### Treatment outcome

**Fig 1:**
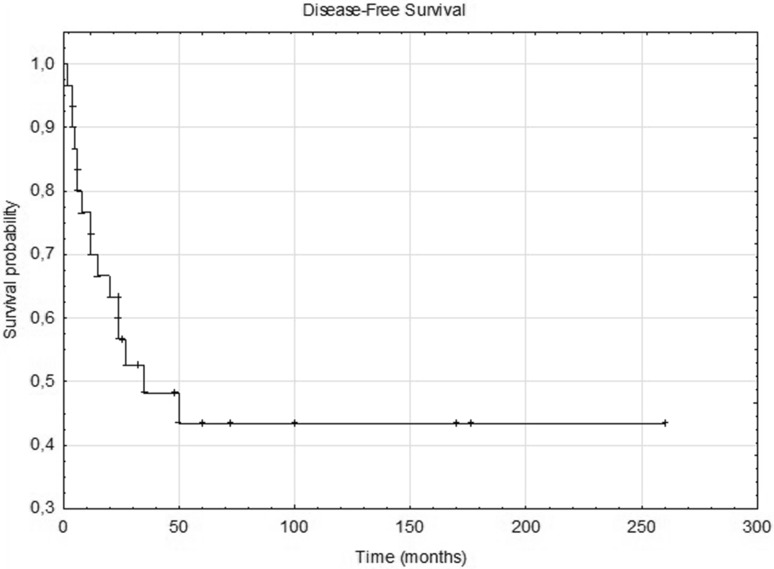
Kaplan–Meier estimate for disease-free survival (DFS). Vertical lines indicate censoring observations

**Fig 2:**
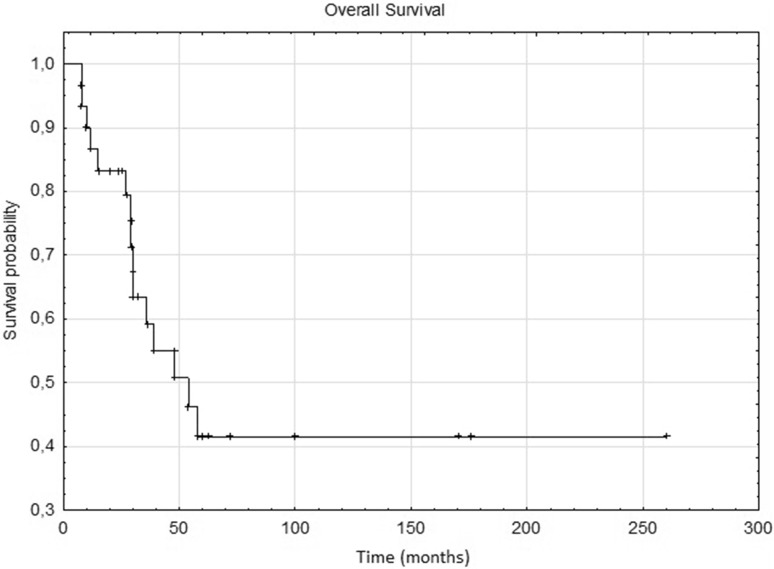
Kaplan–Meier estimate for overall survival (OS). Vertical lines indicate censoring observations

During the follow-up period, the disease recurred in 23 patients (57.5%). Local recurrence concerned 10 patients (25%) and was located: skull base—5 cases, cheek—2 cases, parapharyngeal space—1 case, and in two cases it included the base of the skull, parapharyngeal space, cheek, mandible, and subtemporal fossa. In 15 cases (37.5%), distant metastases (lungs, brain, bones, liver, and non-cervical lymph nodes) were found in the course of observation, including coexisting with local recurrence in two patients and with recurrent neck node in the following two cases. Surgical treatment of local recurrence was possible only in three patients. During the follow-up period, 20 patients (50%) died of cancer, another 4 (16%) of other causes. The remaining patients were without the features of the disease. 5-year disease-free survival (DSF) and overall survival (OS) were 42.5% and 41%, respectively (Figs. 1, 2).

### Prognostic factors

Univariate analysis proved that the significant influence on the 5-year DFS and OS had the following factors: preoperative facial nerve palsy (*p* = 0.024 and *p* = 0.017, respectively), higher pT-stage (*p* < 0.001), radical parotidectomy (*p* = 0.024 and *p* = 0.022), radical treatment of the neck (*p* = 0.001 and *p* = 0.002), EPE (*p* = 0.040 and *p* = 0.028), histology SDCexPA and PNI (*p* = 0.036 and 0.048). These results are summarized in Table 2. Multivariate analysis (Table 3) showed that independent prognostic factors are 7th nerve palsy and histology SDCexPA, which worsened 5-year DFS, respectively, 3.61 and 3.94 times (*p* = 0.033 and *p* = 0.026). On the other hand, on 5-year OS, only 7th nerve palsy had an influence (3.86 times worse prognosis, *p* = 0.033).

**Table 2:** Univariate analysis for 5-year disease-free survival and overall survival

Variables	*N*	Disease-free survival			Overall survival		
		HR	95% CI	*p* value	HR	95% CI	*p* value
Age							
< 55	13	1.00	–	–	1.00	–	–
> 55	27	1.23	0.42–3,61	0.708	1.29	0.47–3.57	0.582
Sex							
Women	17	1.00	–	–	1.00	–	–
Men	23	1.32	0.48–3,65	0.571	1.23	0.45–3.35	0.663
7th nerve (preoperative)							
Normal	17	1.00	–	–	1.00	–	–
Palsy	13	3.18	1.01–10,04	**0.024**	3.03	1.04–8,86	**0.018**
Skin infiltration							
No	24	1.00	–	–	1.00	–	–
Yes	6	1.94	0.59–6.37	0.313	1.47	0.40–5.36	0.584
Other symptoms of malignancy^a^							
No	16	1.00	–	–	1.00	–	–
Yes	24	1.89	0.71–5.05	0.212	1.61	0.60–4.34	0.344
pT-stage							
T1 /T2	10	1.00	–	–	1.00	–	–
T3/T4a	30	NA	-	**0.000**	9.54	3.49–26.07	**0.002**
pN-stage							
*N*-	13	1.00	–	–	1.00	–	–
* N* +	27	1.21	0.43–3.44	0.702	1.19	0.43–3.33	0.727
Resection							
*R*0/*R*0(close margin)	24	1.00	–	–	1.00	–	–
*R*1/*R*2	16	1,35	0,49–3,73	0,552	1,08	0,40–2,91	0,872
Parotidectomy							
Conservative/semiconser.	12	1.00	–	–	1.00	–	–
Radical	28	2.35	0.87–6.60	**0.024**	2.39	0.87–6.60	**0.022**
Neck dissection							
Selective	20	1.00	–	–	1.00	–	–
Radical/modified radical	20	5.51	1.97–15.37	**0.001**	4.59	1.68–12.51	**0.002**
Histology							
SDC	21	1.00	–	–	1.00	–	–
SDC EX PA	19	3.55	1.30–9.73	**0.013**	2.68	0.98–7.34	**0.041**
EPE							
No	8	1.00	–	–	1.00	–	–
Yes	32	NA	–	**0.040**	4.24	1.30–13.83	**0.026**
PNI							
No	15	1.00	–	–	1.00	–	–
Yes	25	3.34	1.24–8.97	**0.036**	2.72	1.02–7.26	**0.048**
Intra/periparotid LN							
pN-	23	1.00	–	–	1.00	–	–
pN +	17	1.38	0.51–3.71	0.514	1.30	0.49–3.47	0.577
HER2 overexpression							
No	27	1.00	–	–	1.00	–	–
Yes	13	1.22	0.41–3.65	0.613	1.28	0.40–3.72	0.680

Bold font indicates statistical significance

*HR* hazard ratio, *CI* confidence interval, *SDCsalivary duct carcinoma,SDC EX PASDC* ex *pleomorphic adenoma*, *EPE* extraparenchymal extension, *PNI* perineural invasion, *LN* lymph nodes, *NA* not applicable, *HER2* human epidermal growth factor receptor 2

^a^Pain and/or tumor rapid grown and/or cervical lymphadenopathy

**Table 3:** Multivariate analysis for 5-year disease-free survival and overall survival

Variables	Disease-free survival			Overall survival		
	HR	95% CI	*p* value	HR	95% CI	*p* value
7th nerve palsy (preoperative)	3.61	1.11–11.81	**0.033**	3.86	1.12–13.32	**0.033**
pT-stage (T1/2 vs. T3/T4)	0.00	0.00	0.996	0.00	0.00	0.997
PNI	3.50	0.69–17.74	0.130	2.68	0.52–13.82	0.238
Histology (SDC vs. SDCexPA)	3.94	1.18–13.15	**0.026**	3.26	0.95–11.22	0.060
EPE	0.28	0.00	1.000	0.46	0.00	1.000

Bold font indicates statistical significance

*HR* hazard ratio, *CI* confidence interval, *PNI* perineural invasion, *EPE* extraparenchymal extension, *SDCsalivary duct carcinoma,SDCexPASDC* ex *pleomorphic adenoma*

## Discussion

According to the literature, SDC develops in adults over a wide age range from 26 to 93 years, however, with a clear average age between 60 and 66 years [4, 7–15]. Male predominates from 66 to 93% [4, 7–10, 12, 15]. In the presented comparison, the average age of patients was 62 years, however, the male predominance was not as pronounced (57.5%).

SDC is characterized by an aggressive clinical course. High stage T3/T4 in the presented series had as much as 75% of patients. In the material of the other authors, stage T3/T4 had from 42 to 66% [7, 8, 10, 14, 15]. Such a large percentage of patients with high T-stage is undoubtedly associated with frequent preoperative VII nerve palsy, which in the presented material occurred in 1/3 of cases. 15% of patients had infiltration and/or skin ulceration qualifying for stage T4. In total, 75% of patients had symptoms indicating the malignancy of the tumor, in addition to the above-mentioned pain (about 40%), rapid tumor growth (35%), and cervical lymphadenopathy (20%). In a multicentre study (141 patients with DSC, including 112 in the parotid gland) presented by Otsuka et al., 30.5% of patients had preoperative nerve palsy, almost 18% had pain, and in about 24% rapid progression of tumor size [14]. In other series, preoperative paresis of the nerve VII was found in 30–50% of patients [8, 11]. Another important factor is the very high rate of the neck lymph node metastases, which were found in 67.5% of patients. These results coincide with data from the literature, as the percentage of patients with neck metastases ranges from 52 to 70% [4, 7, 9, 10, 12]. The above factors cause that the vast majority of patients are treated in the highest IV clinical stage (in this series up to 82.5% and according to other authors from 60.7 to 82% of patients) [4, 9, 11, 12]. Our patients significance is a long time of tumor presence (from 1.5 to 480 months, with a median of 30 months), which is undoubtedly associated with the development of SDC in nearly half of patients in pleomorphic adenoma. In other reports, the percentage of patients with SDCexPA ranged from 20 to 40% [7, 11]. In the case of malignancy symptoms, the time between the symptoms onset and onset of treatment ranged from 1 to 12 months (median 5 months), which is consistent with the observations of the other authors [10].

The high T-stage requires radical treatment with the facial nerve sacrifice. In our material, total radical parotidectomy was performed in 70%, and semiconservative (partial or total with preservation of part of the nerve VII branches) in the next 12.5% of patients. Unfortunately, despite a wide resection, 40% of patients did not have a negative surgical margin, which undoubtedly affected the results of treatment. In other papers, the rate of patients with a resection margin of *R*1 or *R*2 was even higher and ranged from 52 to 62% [11–13].

**Fig 3:**
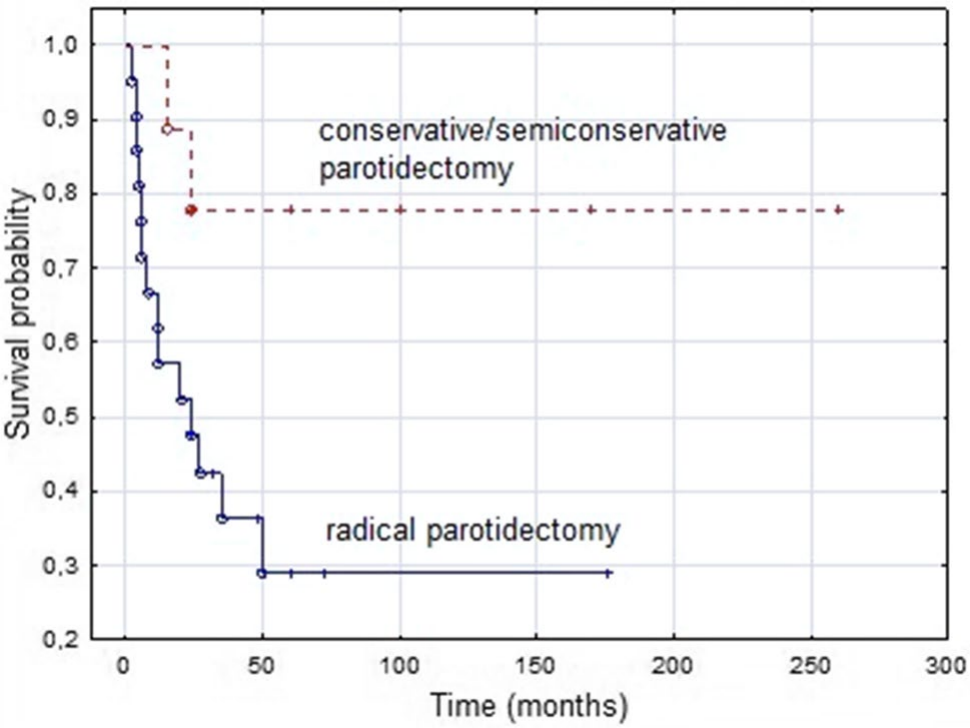
Kaplan–Meier estimate for disease-free survival (DFS) according to the type of parotidectomy. Vertical lines indicate censoring observations

**Fig 4:**
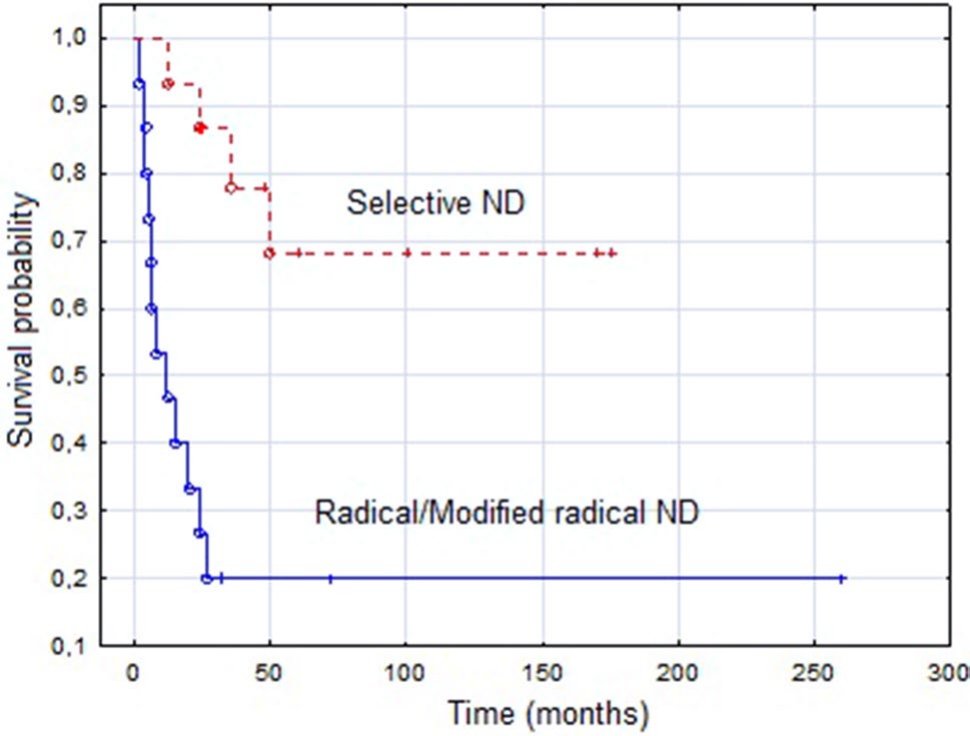
Kaplan–Meier estimate for disease-free survival (DFS) according to the type of neck dissection. Vertical lines indicate censoring observations

The results of the analysis showed statistically significant differences between those, who were treated more and less radically. Patients treated more radically (total radical parotidectomy, radical or modified radical neck dissection) definitely had worse survival because they had much higher local and regional stage of disease (T-stage and N-stage). In our opinion, the effect of type of procedure on the results of treatment can be assessed only by comparing its different variants (e.g., radical vs. conservative parotidectomy) for the same stage (Figs. 3, 4). During the follow-up period, nearly 60% of patients had a relapse. Local recurrence was found in 25% of patients and most often it was referred to the base of the skull, exceeding the indications for rational salvage treatment. In other series, in 9–46% of patients local recurrence was found, without treatment possibilities in most of them [8, 10, 11, 13, 14]. Nearly 40% of patients during follow-up period had metastases distant mainly to the lungs, brain, bones and liver. Also, a high percentage, from 39 to 54% of distant metastases of the SDC was given in other series. In addition to the above locations, they concerned orbits, kidney and spleen [10, 11, 14]. Moreover, in Gilbert’s material, 4% of patients had distant metastases at the time of the diagnosis, and in Osborn et al., this percentage was even higher and amounted to as much as 10% [7, 15]. Distant metastases and inoperative local recurrences, mainly in the skull base, are the most common cause of failure of parotid gland SDC treatment. In the presented series, all patients underwent a neck dissection, half of them radical or modified radical, which undoubtedly contributed to the low rate of nodal recurrences (5%). According to the literature, the nodal recurrence in SDC was found to be about 8–18%, so it is the rarest form of relapse of this disease [8, 10, 11, 14]. During the entire follow-up period, half of the patients died of cancer. Similar results are provided by the other authors [8, 10, 13]. 5-year DSF and OS were just over 40%, which is similar to the one reported by Johnston et al. (OS 43%), while in the material obtained from the Surveillance, Epidemiology, and End Results database (SEER) from the US, 5- and 10-year disease-specific survival (DSS) was 64% and 56%, respectively [4, 11]. Based on the above data, taking into account the high clinical stage of disease in the majority of patients, it can be concluded that despite such aggressive cancer, successful treatment is possible in four out of ten patients. In the analyzed material, the factors significantly influencing the results of treatment (5-year DFS and OS) in the univariate analysis were: preoperative facial nerve paresis, stage pT3/T4, histology SDCexPA, EPE and PNI. The preoperative paresis of the facial nerve (DSF and OS) and histology of SDCexPA (DSF) proved to be an independent prognostic factor. Johnson et al. (2015) in a univariate analysis proved a statistically significant effect of high local stage (pT3/T4), LVI, high regional stage (pN2b/c) and ECS on the OS. Independent prognostic factors for OS were pT3/T4, pN2b/c and ECS. In addition, numerous metastases to the neck lymph nodes (pN2b/c) were an important prognostic factor for distant metastases [11]. In the large multicenter series from Japan in 2016 presented by Otsuka et al. (141 patients, 112 parotid glands), worse OS were obtained in patients over 65 years of age, with histologically confirmed lymph node metastases (pN + ) and rapid progression of the tumor. Age and pN1/2 proved to be independent prognostic factors for OS in multivariate analysis. On the other hand, in addition to rapid progression of the tumor and high-grade of pN, the presence of pain had a significant impact on DFS [14]. In the paper of Gilbert et al., from 2016, with 75 SDC cases of large and small salivary glands (including 62 parotid glands), OS was influenced by pT-stage, ECS, facial nerve sacrifice, while DFS was influenced by ECS and PNI. In multivariate analysis for worse OS, statistical significance was demonstrated for age and high nodal stage (pN2/3) and for DFS—male gender. It is also noteworthy that these authors did not show statistically significant differences in DFS and OS between SDC de novo and ex pleomorphic adenoma [7]. On the basis of the largest published SDC series, based on the National Cancer Database from USA, 495 SDC cases (including 396 on the parotid location), it was shown that the independent negative prognostic factors affecting the OS are the increasing age, male gender and stage IVa and IVc. It is also worth emphasizing that this study did not show the influence of adjuvant RT or chemoradiotherapy on OS [15]. In the material of Han et al., from 2015 (28 cases of SDC, including 20 in the parotid gland) in the univariate analysis, it was found that advanced N classification (pN2b), ECS and PNI have a statistically significant effect on 3-year DFS and distant metastasis-free survival (DMFS), while 3-year OS was influenced by LVI and PNI. Independent negative prognostic factors for 3-year DFS and DMFS were pN2b and PNI and OS—LVI and PNI [9]. In a similar to the presented series of 38 patients with SDT parotid by Shi et al., from 2014, independent negative factors affecting recurrence-free survival (RFS) turned out to be pN + , postoperative radiation, facial nerve palsy according to House–Brackmann grade. On the other hand, the male gender, pN + , higher stage, nerve invasion, and preoperative House–Brackmann grade also had a negative impact on the OS, however, no statistical significance was found in multivariate analysis regarding gender and stage [8]. In a very large series of 228 SDC cases (including 163 in parotid gland) based on the SEER database presented by Jayaprakash et al. (2014), it was shown that younger age, lower grade, early stage I/II, lack of metastases to the neck lymph nodes (pN-), and smaller tumors ≤ 3 cm had a statistically significant positive effect on DSS and OS. Treatment type (surgery only) had a significant impact on OS and DSS only in univariate analysis. It was also found that there is a significant linear relationship between size and likelihood of lymph node involvement, with better prognosis for patients with smaller tumor and pN + than with a larger tumor and pN-. However, despite the size of this series, it has some limitations because we analyzed also cases of low grade SDC, which is a completely different carcinoma and in the current WHO classification is called intraductal carcinoma [4]. In summary, the most common independent prognostic factors in SDC were histologically confirmed metastases to the neck lymph nodes (pN + )—6 series and a higher age (4 series) [4, 7–9, 11, 14, 15]. A summary of negative prognostic factors in the literature is presented in Table 4.

**Table 4:** Negative prognostic factors in SDC according to literature

Series (reference) year	Present (2018)	15 (2017)	14 (2016)	6 (2016)	10 (2015)	8 (2015)	7 (2014)	4 (2014)
No of pts (parotid)	40	495 (396)	141 (112)	75 (62)	54 (49)	28 (20)	38	228 (163)
Negative prognostic factor								
Higher age		m	m	m				m
Male gender		m					u	
T3/T4	u			u	m			m
pN +			m	m	m	m	m	m
Stage IV		m					u	m
ECS					m	u		
EPE	u			u				
PNI	u			u		m		
LVI					u	m		
Facial palsy	m						m	
Pain			u					
Rapid progression			u					
SDCexPA	m							
PORT							m	u

*ECS* extracapsular spread, *EPE* extraparenchymal extension, *PNI* perineural invasion, *LVI* lymphovascular invasion, *SDCexPASDC* ex pleomorphic adenoma, *PORT* postoperative radiotherapy, *U*, *M* statistical significance in u—univariate and *m*—multivariate analyses

In our material, about one-third of patients had overexpression of HER2 but without the significant influence on the 5-year DFS and OS. In the literature, this percentage varies from 26% to over 40% [7, 16, 17]. In the study of Jaehne et al., expression of HER2 correlated with early local recurrences, distant metastases and survival rates [10]. However, in other studies, the effect of this marker on survival was not found and its relationship to parameters such as age, sex, T- and N-stages [7, 17] was not demonstrated. In addition to HER2 in SDC, expression of other proteins such as the *androgen receptor* (AR), Ki-67 and *epidermal growth factor receptor* (EGFR) is determined. Especially the latter protein raises hopes as a potential prognostic factor and target for therapy. It is also important that HER2 and EGFR, although they belong to this family, are markers independent of each other [17]. Masubuchi et al. proved that EGFR is an independent predictor of DFS, but not affecting OS [16]. In the material of Williams et al., EGFR expression was high (70%) and correlated statistically significantly with local recurrence and occurred more frequently, but without statistical significance in patients with distant metastases and with poor 3-year survival [17]. Han et al. did not show any relationship between EGFR expression and recurrences and distant metastases [9]. Although the role of molecular status as a prognostic factor in patients with SDC is debatable, the search for novel genetic changes in SDC in the future may extend therapeutic options in patients with unresectable, metastatic or recurrent tumors [12, 16].

## Conclusions

SDC is a clinically aggressive cancer with a high risk of distant metastases, but with a chance of successful cure of around 40%. In the majority of patients, a radical surgical treatment is necessary due to the high degree of clinical stage. Worse prognosis is observed in patients with preoperative facial nerve palsy and in whom SDC develops in pleomorphic adenoma.
